# Active-Learning-Guided Acoustic Metamaterial Resonators for Low-Frequency Noise Suppression and Piezoelectric Energy Harvesting

**DOI:** 10.3390/mi17060685

**Published:** 2026-05-31

**Authors:** Syed Muhammad Anas Ibrahim, Jungyul Park

**Affiliations:** 1 Department of Mechanical Engineering, Sogang University, 35 Baekbeom-ro (Sinsu-dong), Mapo-gu, Seoul 04107, Republic of Korea; anas.ibrahim2011411@gmail.com; 2 Research Institute for Smart Design & Manufacturing Technology, Sogang University, 35 Baekbeom-ro (Sinsu-dong), Mapo-gu, Seoul 04107, Republic of Korea

**Keywords:** acoustic metamaterial, resonator, energy harvesting, noise barrier, active learning

## Abstract

Low-frequency traffic noise below 500 Hz is difficult to mitigate because its long wavelengths require impractically large conventional resonators. Here, we report an active-learning-guided inverse-design approach for scalable phononic-crystal-based acoustic metamaterial resonators that simultaneously suppress low-frequency noise transmission and harvest acoustic energy. The approach combines Gaussian process regression surrogate modeling with genetic algorithm optimization to efficiently explore high-dimensional cavity geometries. By iteratively retraining the surrogate with FEM-validated designs, the active-learning process guides the search toward high-performance structures while reducing costly FEM evaluations compared with conventional GA optimization. After geometric scaling, the 2.5D prototype derived from the nine-point optimized cavity achieved a pressure amplification factor of approximately 20 near 490 Hz, while the revolved 3D cavity exhibited amplification exceeding 30 and a transmission loss of approximately 14 dB near the target frequency. Integrated with a mass-loaded five-PZT stack, the device generated 5.5 V_pp_ and 0.25 mW under 100 dB SPL, corresponding to a normalized power density of 0.58 μW Pa^−2^ cm^−3^. These results demonstrate a route toward multifunctional piezoelectric acoustic devices for noise mitigation, localized energy harvesting, and self-powered sensing.

## 1. Introduction

With rapid urbanization, traffic noise containing strong low-frequency components around 500 Hz has become a serious environmental problem that degrades the health and quality of life of urban residents. Beyond its adverse impact as a noise pollutant, traffic noise also represents a pervasive and largely untapped source of ambient acoustic energy [1,2]. Such acoustic energy is continuously generated in traffic corridors, railway systems, tunnels, and industrial facilities, making it attractive for localized energy harvesting applications [3,4,5]. In contrast, conventional renewable energy harvesters, including solar and wind systems, are strongly constrained by environmental conditions and installation sites. For example, in underground malls, subway stations, and enclosed indoor spaces where solar irradiation and wind flow are limited, acoustic energy harvesting offers a complementary route for powering distributed sensors and low-power monitoring devices [6,7,8,9]. Accordingly, the development of acoustic devices that can both mitigate unwanted noise and harvest otherwise wasted acoustic energy offers a promising strategy for multifunctional and sustainable urban infrastructure.

In recent years, acoustic energy harvesting systems have been extensively investigated through various structural and material innovations. Rectangular Helmholtz resonators integrated with piezoelectric materials have been widely used to convert acoustic energy into electrical power [10,11], while magnetoelastic soft-matter systems have also been exploited for acoustic energy harvesting [1]. Low-frequency acoustic focusing has been enhanced by combining 1-bit coding metasurfaces with triboelectric nanogenerators (TENGs) [12], and trampoline metamaterials coupled with Helmholtz resonators have been proposed to improve piezoelectric harvesting efficiency [13]. Tunable PZT circular plates have also been developed as low-frequency acoustic harvesting barriers for subway tunnels based on optimized Helmholtz resonators [14]. In addition, TENG-based systems with various structural configurations have been designed to enhance output performance [15,16,17]. Other structural approaches include vertical cantilever beams embedded in Helmholtz resonators with canonical or conical necks [5], ultrathin mechanically rigid metasurface absorbers [18], and spiral-shaped cavities for improved energy capture [11]. Acoustic gratings integrated with piezoelectric plates have been shown to generate broadband voltage output [19], while nonlinear piezoelectric harvesting using bistable shakers has also been investigated [20]. Furthermore, composite materials, active acoustic metasurfaces, graded resonator arrays, bidirectional resonators, unidirectional collection modules, and multilayer configurations have been explored to improve collection efficiency, operational bandwidth, and overall harvesting capacity [21,22,23,24,25,26,27,28].

Despite these advances, most phononic crystal- and resonator-based acoustic harvesters still rely on geometrically periodic or manually parameterized cavity designs. Such geometrically homogeneous structures provide limited freedom to tailor local pressure distributions, particularly at low frequencies where long wavelengths require bulky resonator dimensions and the design space involves many strongly coupled geometric variables [28]. Recently, artificial intelligence (AI) and machine learning (ML) techniques have been increasingly used to model complex relationships between high-dimensional design parameters and physical responses across a range of technologically important system [29,30,31,32,33,34,35]. By leveraging the rapid inference capability of ML models, optimization problems involving microstructure and shape design have been successfully addressed in areas such as mechanical property enhancement [36,37], thermal transport optimization [38], inverse design and analysis of metamaterials [39,40], and electromagnetic response tuning [41]. In acoustic and phononic systems, ML-based inverse design can map target responses, such as desired frequencies or dispersion characteristics, to corresponding physical design parameters [42]. However, experimentally validated active-learning-guided design approaches for spatially heterogeneous acoustic cavities capable of simultaneous pressure amplification, transmission suppression, and piezoelectric energy harvesting remain underexplored.

**Figure 1:**
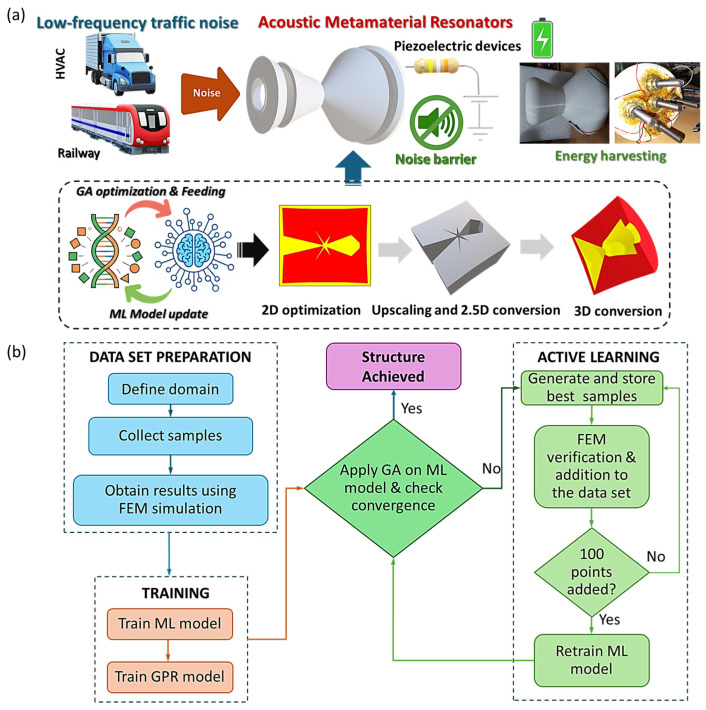
(**a**) Schematic and (**b**) process flow of designing acoustic metamaterial resonator using active learning (AL)-driven optimization process for low-frequency noise suppression and piezoelectric energy harvesting.

In this study, we optimize a phononic-crystal (PnC)-based acoustic cavity to maximize pressure amplification from low-frequency traffic noise near 500 Hz using an iterative ML-assisted inverse-design approach integrated with genetic algorithm (GA) optimization (Figure 1). Although a standalone GA can search for high-performance geometries, it requires substantial computational cost because each candidate design must be evaluated using finite element analysis. To address this limitation, we employ a Gaussian process regression (GPR) surrogate model to approximate the relationship between cavity geometry and the resulting internal pressure field. The trained surrogate enables rapid evaluation of candidate geometries, while GA-based optimization generates new cavity designs with enhanced pressure amplification. Unlike static surrogate models, the GPR model is iteratively updated with newly FEM-validated designs, thereby improving prediction accuracy and guiding the search toward high-performance regions of the design space through active learning. This strategy substantially reduces expensive FEM evaluations while preserving the ability to discover complex cavity geometries with strong acoustic amplification and transmission suppression.

The proposed system consists of two coupled functional modules: an active-learning-designed acoustic amplification module and a piezoelectric energy conversion module. Incoming acoustic waves are concentrated and amplified inside the optimized cavity near the target resonance frequency. The amplified acoustic energy is then converted into electrical output using PVDF films and PZT-based harvesters positioned near the pressure-amplified region. To validate the design experimentally, optimized 2D cavity geometries were converted into 2.5D extruded and 3D revolved structures and fabricated using 3D printing. Experimental measurements confirmed that placing the PVDF film or PZT element at the location of maximum pressure amplification significantly enhanced the electrical output, in agreement with the numerical predictions. These results demonstrate that acoustic energy harvesters embedded within noise-suppressing metamaterial structures can provide a practical route toward localized power generation for self-powered sensing and monitoring applications in high-noise environments.

## 2. Materials and Methods

### 2.1. Numerical Model and Design Domain Definition

**Figure 2:**
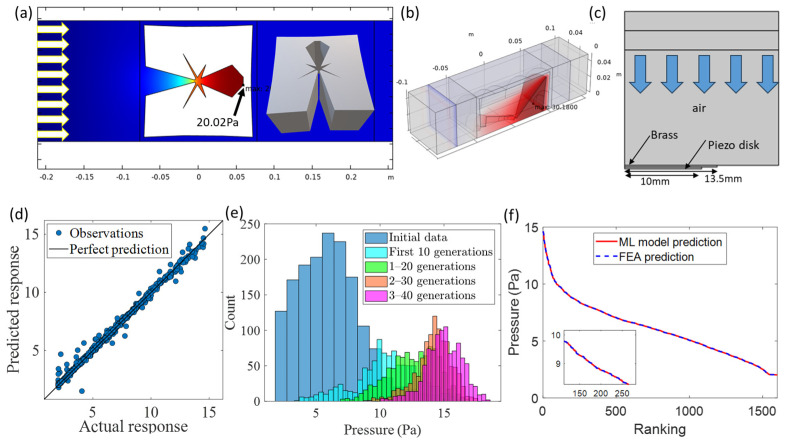
Numerical simulation of acoustic metamaterial resonators and performance evaluation of the GPR surrogate model. (**a**) Two-dimensional unit-cell simulation domain (22 × 22 mm^2^) with periodic boundaries and perfectly matched layers (PMLs). (**b**) Revolved 3D geometry used for experimental validation. (**c**) Schematic of the piezoelectric coupling simulation. (**d**) Regression plot comparing GPR-predicted and FEM-computed acoustic responses, showing high predictive accuracy with R^2^ = 0.98. (**e**) Evolution of pressure-amplification distributions over successive active-learning generations, showing a shift toward higher-performance designs. (**f**) Ranking curve showing close agreement between GPR surrogate predictions and FEM ground-truth results.

Numerical simulations were performed using finite element analysis (FEA) in COMSOL Multiphysics v6.3. For modeling the resonator structure, we used pressure acoustic and thermoviscous modules. In addition, the PZT energy harvester was modeled by coupling the Solid Mechanics and Electrostatics module. To reduce computational cost during the primary optimization stage, the cavity was first modeled in a two-dimensional domain and subsequently converted into 2.5D extruded and 3D revolved structures for experimental validation and energy harvesting analysis (Figure 2a and Figure S2). The square unit cell had an initial side length of $a_{1}=22$ mm, which was later scaled to tune the resonance frequency to the desired range. To approximate an open acoustic environment, perfectly matched layers (PMLs) were applied at the horizontal boundaries of the computational domain to absorb outgoing waves and suppress artificial reflections. We have used the sound hard boundary condition at the transverse boundaries. Mainly, we used triangular and tetrahedral elements for capturing the 2D and 3D geometries, respectively. To assure the fidelity of simulation results, the maximum element size was kept less than λ/6 (see Figure S5). A plane acoustic wave was applied at the inlet as an excitation source.

The cavity boundary was parameterized using radial control points inside the unit cell. Each boundary point was defined by the polar coordinates $\left(r_{i},\;\theta_{i}\right)$ and converted into Cartesian coordinates as $x_{i}=r_{i}cos\theta_{i}$, $y_{i}=r_{i}sin\theta_{i}$, where $r_{i}$ is the radial distance of the i-th control point and $\theta_{i}$ is its angular position. The radial distance was constrained within predefined lower and upper bounds as

(1) $$r_{i}\in \left[\frac{a_{1}}{20},\frac{cos\left(\frac{\pi }{n}\right)\frac{a_{1}}{2}}{cos\left(\theta_{i}-\frac{2\pi }{n}.\left\lfloor \frac{n\theta_{i}+\pi }{2\pi }\right\rfloor \right)\sin\left(\theta_{i}\right)}-\frac{a_{1}}{10}\;\right]$$

where n defines the number of polygonal sectors used to constrain the cavity boundary. For the nine-point design case, the angular positions were assigned as $\theta_{i}=[5{}^{\circ},25{}^{\circ},45{}^{\circ},65{}^{\circ},105{}^{\circ},120{}^{\circ},135{}^{\circ},150{}^{\circ},165{}^{\circ}]$ for i∈(1,9). By varying the radial distances within these bounds, different cavity geometries with controlled geometric complexity were generated. In the resonator design we consider allocating space for the spatial allowance of inlet while maintaining vertical symmetry and minimizing the geometric degrees of freedom (control points). By increasing the angular resolution, expanding the unit cell design space exploration, it inherently compromises computational efficiency. To balance this trade-off, a discretized parametric sweep is implemented: the first quadrant utilizes four points initiating at 5° with a 20° step size, while the second quadrant incorporates five points ranging from 105° to 165° at a 15° increment. This geometric parameterization yields a versatile topology capable of transitioning continuously from circular to square cavity profiles as shown in Figure S1a.

Preliminary optimization studies targeted frequencies of 2.5, 3.0, 3.5, and 4.0 kHz using cavity geometries defined by four, six, and nine radial control points. The initial objective was to absorb sound and harvest acoustic energy in human speech spectrum (300–3500 Hz). However, due to the exceptionally low acoustic power density of conversational speech within this band, the available incident energy was insufficient to efficiently drive the harvesting mechanism. To overcome this limitation, the target operational bandwidth was shifted to the low-frequency regime (≤500 Hz), by leveraging the upscaling property and thereby maximizing the power output.

In the final optimization procedure, these radial distances served as the design variables, and the objective function was defined as the maximization of the acoustic pressure generated inside the cavity. To reduce the dimensionality of the 3D design problem, the revolved 3D structure was designed with vertical and horizontal symmetry, as shown in Figure 2b. The PZT-based energy harvester was then simulated under the experimentally measured pressure field inside the cavity to predict the voltage output (Figure 2c). To model the electromechanical conversion of the PZT disc under the internal acoustic field (Figure 2c), a fully coupled multiphysics interface combining pressure acoustics, solid mechanics, and electrostatics was solved. The forward coupling transfers the localized acoustic pressure field p as a normal mechanical boundary load $F_{A}=p\cdot n$ across the face of the PZT disc. The structural deformation and subsequent charge generation within the piezoelectric material are governed by the linear constitutive equations in stress-charge form:

(2) $$T=c_{E}S-e^{T}E$$

(3) $$D=eS+\varepsilon_{S}E$$

where T is the stress tensor, S is the strain tensor, E is the electric field vector, D is the electric displacement vector, $c_{E}$ is the elasticity matrix (at constant electric field), e is the coupling matrix, and $\varepsilon_{S}$ is the permittivity matrix (at constant strain). Figure 2c shows the asymmetric design of the design domain, where pressure is incident from the top towards the PZT disc.

### 2.2. Gaussian Process Regression Surrogate Modeling

To capture the nonlinear relationship between radial cavity coordinates and acoustic pressure amplification, a Gaussian process regression (GPR) surrogate model was constructed. The model employed a Matérn 5/2 kernel, which was selected together with its hyperparameters through an internal Bayesian optimization routine during training. This kernel was chosen because it effectively captures smooth but nonlinear variations in the acoustic response.

The initial training dataset consisted of 1600 cavity design combinations generated from the radial coordinate design space. To evaluate the robustness of the model for the available dataset size, 20-fold cross-validation was performed. As shown in Figure 2d, the FEM-computed pressure amplification values were compared with the GPR-predicted values using the coefficient of determination, $R^{2}=0.98$. The data points were closely distributed along the y=x line, indicating high predictive accuracy.

### 2.3. Active Learning and Evolutionary Optimization

The optimization was performed using an iterative surrogate-assisted genetic algorithm (GA). In this process, each candidate cavity geometry was represented as a chromosome, and each gene encoded a radial distance coordinate defining the cavity boundary. For each candidate geometry, the GPR surrogate predicted both the mean pressure amplification, μ(x), and the predictive uncertainty, σ(x).

To balance exploitation of high-pressure designs and exploration of uncertain regions, candidate geometries were ranked using an upper-confidence-bound acquisition function: α(x)=μ(x)+κσ(x),  where κ controls the relative contribution of uncertainty-driven exploration. The optimization objective was therefore formulated as

(4) $$\begin{array}{cccc} & x^{*}=arg\;\underset{x_{i}^{\left(K\right)}}{max}\;\alpha \left(x_{i}^{\left(K\right)}\right), & & \end{array}$$

where $x^{*}$ is the optimal design, $x_{i}^{\left(K\right)}$ denotes the i-th candidate geometry in the K-th generation, and α is the acquisition function derived from the GPR surrogate.

New generations were produced through crossover, in which a division point was randomly selected between parent chromosomes, and mutation, in which selected genes were randomly perturbed within the prescribed radial bounds. In each generation, the candidate population was evaluated using the GPR surrogate, and the top 100 candidates with the highest acquisition values were selected for high-fidelity FEM validation. The resulting FEM-computed pressure amplification values were treated as ground-truth data and appended to the training dataset. The GPR model was then retrained using the updated dataset before the next active-learning cycle, as illustrated in Figure 2e and Figure S1b.

This iterative GPR-assisted optimization process was repeated until convergence was achieved, substantially reducing the number of costly FEM evaluations compared with the conventional GA baseline. The final surrogate predictions showed good agreement with the FEM-validated results, as shown in Figure 2f.

## 3. Results and Discussion

### 3.1. Geometric Evolution and Frequency Dependence

To explore the design space, cavity geometries defined by four, six, and nine radial control points were optimized at target frequencies of 2.5, 3.0, 3.5, and 4.0 kHz. The optimized structures and their corresponding pressure amplification factors are summarized in Table 1.

The results reveal a clear dependence of amplification performance on geometric complexity. The four-point cavities showed the lowest pressure amplification because their limited design freedom restricted the formation of resonant internal features. By contrast, the nine-point geometries consistently yielded the highest amplification factors, suggesting that increased geometric flexibility enables more effective pressure localization and acoustic resonance within the cavity.

In addition, the frequency-dependent pressure response varied with the number of radial control points. For the four-point and six-point cavities, the amplification factor tended to decrease with increasing target frequency. Conversely, the nine-point geometries showed an increasing trend, with higher target frequencies generally leading to stronger pressure amplification. Geometries with flat bottoms or insufficient boundary variation produced weak pressure responses, emphasizing the need for optimized spatially heterogeneous cavity topologies to achieve strong acoustic amplification.

### 3.2. Convergence Analysis: Standard GA vs. Active Learning

**Figure 3:**
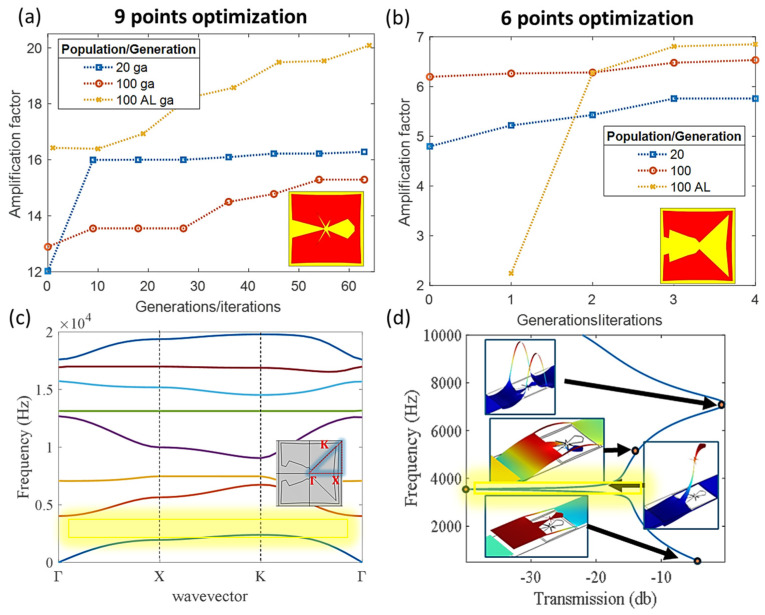
Optimization convergence and physical characterization of the evolved metamaterial. (**a**) Convergence history for the nine-point geometry, showing that the AL routine (yellow dotted line) achieves a higher amplification factor (∼20.1) significantly faster than standard GA with varying population sizes. (**b**) Convergence history for the six-point geometry, confirming the superior efficiency of the AL framework. (**c**) Band structure diagram of the optimized unit cell, exhibiting a complete acoustic bandgap (yellow shaded region) around the target frequency. (**d**) Pressure as a height function at different frequencies at different location of transmission curve.

The computational efficiency of the proposed AL + GA strategy was benchmarked against conventional GA optimization. Figure 3a,b shows the convergence behavior for the nine-point and six-point geometries, respectively. In the nine-point optimization space, the geometric boundaries possess high-dimensional degrees of freedom, creating a highly non-linear, non-convex optimization landscape filled with numerous deceptive local optima. A conventional GA searches blindly by performing expensive FEM evaluations across entire populations. When the population size is small (e.g., 20), it lacks genetic diversity and stalls early. When the population is large (e.g., 100), it demands an impractical number of iterations and is frequently trapped in local extrema. The proposed framework utilizes surrogate model combined with the upper-confidence-bound acquisition function: $\alpha \left(x\right)=\mu \left(x\right)+\alpha \sigma \left(x\right)$. The parameter α balances exploitation (targeting regions where the mean predicted pressure amplification $\mu \left(x\right)$ is maximum) and exploration (targeting regions where model uncertainty $\sigma \left(x\right)$ is high). This allows the algorithm to rapidly bypass unpromising design topologies without wasting computational resources, achieving a higher pressure amplification factor (~20.1) with fewer costly FEM simulations.

The optimized structures also exhibited an absolute bandgap near the target frequency, indicating their potential for acoustic transmission suppression (Figure 3c,d). This bandgap arises from local resonance where incoming sound is absorbed. Precisely at the resonance frequency within the bandgap, incoming wave energy cannot transmit forward; instead, it becomes heavily localized, confined, and strongly amplified inside the asymmetric cavity.

As summarized in Table 2, the AL + GA strategy achieved the same amplification factor as conventional GA with tenfold fewer FEM evaluations in the six-point case. For the nine-point case, AL + GA achieved a higher amplification factor of 20.1 using fivefold fewer FEM evaluations, whereas conventional GA converged to lower values of approximately 16–18 even after 300 generations.

The convergence behavior was also affected by population size. For six-point geometries, increasing the population size improved the maximum converged pressure amplification. In contrast, for nine-point geometries, larger populations in conventional GA did not consistently improve performance, likely because the more complex design space increased the risk of convergence to local optima. These results demonstrate that the AL-guided surrogate strategy can more efficiently explore complex cavity design spaces than conventional GA.

### 3.3. Selection of the Optimal Design

Based on the optimization study, the nine-point geometry optimized at 3.5 kHz was selected for the 2.5D extruded prototype because it achieved the highest-pressure amplification factor of approximately 20.1 and retained sufficient bottom space for piezoelectric harvester integration. To preserve this integration space, the first five radial points were fixed at their maximum values, while the remaining four points were allowed to evolve. This design constraint maintained strong acoustic pressure amplification while enabling the placement of a PVDF film or PZT element at the cavity bottom, as indicated by the yellow structures in Table 1.

In contrast, the revolved 3D cavity was generated from the six-point optimized geometry because the more complex nine-point geometry posed fabrication challenges during 3D conversion. The 3D configuration was used to evaluate acoustic confinement and integration with PZT-based harvesters in a manufacturable geometry.

### 3.4. Experimental Verification

#### 3.4.1. Fabrication and Experimental Setup

To validate numerical optimization based on active-learning-guided inverse-design, physical prototypes of the 2.5D and 3D cavities were fabricated from polylactic acid (PLA) using a Bambu Lab A1 mini 3D printer. The optimized geometry, originally designed at 3.5 kHz, was spatially upscaled to reduce the resonance frequency to approximately 500 Hz, thereby targeting low-frequency environmental noise. This scaling was based on the inverse relationship between resonance frequency and characteristic geometric dimensions [43].

**Figure 4:**
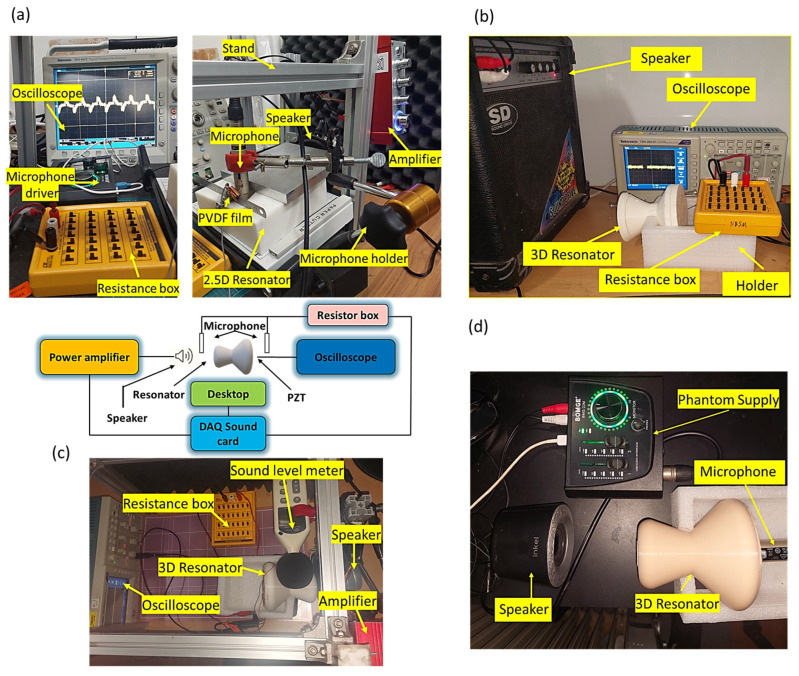
Experimental setups for validation of the active-learning-guided inverse design. Photographs and schematics of the measurement configurations used for energy harvesting and acoustic characterization: (**a**) 2.5D resonator integrated with a PVDF film, (**b**) 3D resonator integrated with a PVDF film, (**c**) 3D resonator integrated with a PZT disc for high-power energy harvesting, and (**d**) pressure-monitoring setup used to quantify acoustic performance, including transmission loss and pressure amplification factor.

The experimental characterization included four configurations, as shown in Figure 4: (i) a 2.5D cavity with a PVDF film for initial pressure and energy harvesting measurements, (ii) a revolved 3D cavity with a PVDF film, (iii) a 3D cavity integrated with a PZT disc for enhanced power output, and (iv) a pressure-monitoring setup for quantifying transmission loss and internal acoustic pressure amplification.

Input acoustic waves were generated using a loudspeaker driven by a Kinter MA-180 power amplifier. An in-house MATLAB R2024a code is used to generate frequency-sweep signals, which are transmitted by the sound card to speaker (C-77B10K Sammi 3-inch full range from Sammi Sound Tech Co., Ltd., Gumi, Republic of Korea and 5 W, Inkel Corp., Incheon, Republic of Korea).For energy harvesting characterization, the output voltage was recorded using digital storage oscilloscopes (TDS 2012B, Tektronix Inc., Beaverton, OR, USA and DPO 4032, Tektronix Inc., Beaverton, OR, USA), as shown in Figure 4a,c. The incident sound pressure level was measured by a digital sound level meter (GM1335, Benetech, Shenzhen, China), and the electrical loading was controlled by a variable resistance box (RBOX-408, Lutron Electronic Enterprise Co., Ltd., Taipei, Taiwan). The acoustic response was captured using condenser microphones (C-2, Behringer, Willich, Germany) powered by an audio interface module with a 48 V phantom power supply (BMG22, BMG Korea, Seoul, Republic of Korea) as shown in Figure 4d.

#### 3.4.2. Acoustic Performance and Model Validation

The pressure amplification factor, A, was experimentally calculated as $A=\frac{V_{w}}{V_{w/o}},$ where $V_{w}$ and $V_{w/o}$ denote the microphone voltage responses measured with and without the cavity, respectively, under identical input conditions. To adapt the optimized design for operation in the traffic-noise regime near 400 Hz, geometric scaling was applied to the optimized cavity structures. The scaling factor for the 2.5D structure, $\zeta_{2.5D}$, was determined as $\zeta_{2.5D}=\frac{f_{2D}^{opt}}{f_{2.5D}^{des}},$ where $f_{2D}^{opt}$ is the optimized resonance frequency of the original 2D design and $f_{2.5D}^{des}$ is the desired resonance frequency after scaling. For operations near 400 Hz, the scaling factor was set to approximately seven. To enable practical implementation, the optimized 2D cavity was first extended along the z-direction to form a 2.5D configuration.

In addition, a revolved 3D cavity was generated by rotating the optimized geometry around the x-axis. This 3D conversion reduced the resonance frequency of the original six-point design to $f_{3D}^{opt}=1840$ Hz. The 3D cavity was then upscaled by a factor of $\zeta_{3D}=\frac{f_{3D}^{opt}}{f_{3D}^{des}}=4.6,$ to align its resonance frequency with the target frequency of $f_{3D}^{des}$ ≤ 500 Hz.

**Figure 5:**
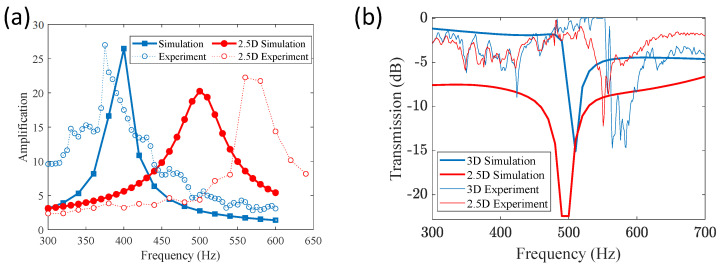
Experimental validation of the FEA simulations for the optimized acoustic metamaterial resonators. (**a**) Comparison of simulated and experimentally measured pressure amplification factors for the 2.5D and 3D cavities. The circle and square markers indicate the 2.5D and 3D cases, respectively, showing good agreement between simulation and experiment and high pressure amplification near the target frequency. (**b**) Simulated and experimentally measured transmission spectra of the 2.5D and 3D structures, demonstrating acoustic transmission suppression near the resonance frequency.

As shown in Figure 5a, the pressure amplification factors of the 2.5D and 3D cavities were compared using both numerical simulations and experimental measurements. After geometric scaling, the 3D cavity prototype achieved a pressure amplification factor of approximately 30, whereas the 2.5D cavity showed amplification factors of approximately 20 in both simulation and experiment. The revolved 3D cavity yields a noticeably higher pressure amplification factor (~30) compared to the 2.5D extruded configuration (~20). This occurs because revolving the 2D optimized profile into a 3D geometry creates an enclosed omnidirectional focus. The incoming acoustic wavefront is compressed symmetrically across all radial planes into a singular central hotspot, whereas the 2.5D structure only compresses waves along a single transverse axis.

The lower resonance frequency of the 3D configuration compared with the corresponding 2D/2.5D structure can be explained by the increased effective acoustic volume of the revolved geometry.

This frequency reduction can be understood using a simplified Helmholtz-type resonance model. For a 2D C-shaped cavity, assuming a unit out-of-plane depth, the resonance frequency can be approximated as

(5) $$\begin{array}{cccc} & \begin{array}{cccc} & f_{2D}=\frac{c}{2\pi }\sqrt{\frac{S_{n,2D}}{V_{2D}L_{n}}}=\frac{c}{2\pi }\sqrt{\frac{x}{\pi r^{2}L_{n}}}, & & \end{array} & & \end{array}$$

where c is the speed of sound, $S_{n,2D}=x$ is the effective neck opening per unit depth, $V_{2D}=\pi r^{2}$ is the effective cavity area per unit depth, r is the effective cavity radius, x is the neck width, and $L_{n}$ is the effective neck length.

For the revolved 3D cavity, the neck opening and cavity volume can be approximated as $S_{n,3D}=\pi x^{2}/4$ and $V_{3D}=4\pi r^{3}/3$, respectively. Here, x is treated as the characteristic neck width in the 2D model and as the effective neck diameter after revolution in the 3D approximation. The corresponding resonance frequency is therefore given by

(6) $$\begin{array}{cccc} & \begin{array}{cccc} & f_{3D}=\frac{c}{2\pi }\sqrt{\frac{S_{n,3D}}{V_{3D}L_{n}}}=\frac{c}{2\pi }\sqrt{\frac{\pi x^{2}/4}{(4\pi r^{3}/3)L_{n}}}. & & \end{array} & & \end{array}$$

Comparing Equations (3) and (4) gives

(7) $$\begin{array}{cccc} & \begin{array}{cccc} & \frac{f_{2D}}{f_{3D}}=\sqrt{\frac{16r}{3\pi x}}. & & \end{array} & & \end{array}$$

Because the effective cavity radius r is larger than the neck width x in the present cavity design, Equation (5) indicates that $f_{2D}>f_{3D}$. This simplified scaling analysis explains why revolving the 2D cavity into a 3D configuration lowers the resonance frequency, which was also confirmed by FEA simulations prior to geometric scaling.

The experimental results showed good agreement with the numerical predictions, with maximum pressure amplification occurring near the target resonance frequency of approximately ≤500 Hz. A slight frequency deviation of approximately 20–25 Hz was observed between the simulated and experimental peaks, which can be attributed to environmental noise, temperature variations, material-property deviations, fabrication tolerances, and microphone positioning. The experimental response was also broader than the idealized simulation, likely because of acoustic losses and imperfect boundary conditions in the measurement setup. In addition to pressure amplification, transmission analysis showed that the optimized structure achieved a transmission loss of approximately 14 dB near the target frequency. This result confirms that the proposed resonator can simultaneously concentrate acoustic energy for harvesting and suppress transmitted sound, as shown in Figure 5b.

### 3.5. Energy Harvesting Characterization

This section describes the energy harvesting characterization of the optimized acoustic resonators. Two types of piezoelectric harvesters were evaluated: a PVDF film cantilever and PZT discs. The PVDF film and PZT disc were bonded to the bottom of the cavity, where strong pressure amplification was observed. Details of the assembled configuration are provided in Figure S3.

#### 3.5.1. PVDF Cantilever: Proof-of-Concept Energy Harvesting

Initial tests were performed using a piezoelectric PVDF cantilever (LDT-028, TE Connectivity Ltd., Hampton, VA, USA) with dimensions of 4.1 mm in length, 1.6 mm in width, and 0.2 mm in thickness. The cantilever was positioned at the base of the cavity to enhance its interaction with the amplified acoustic pressure field.

**Figure 6:**
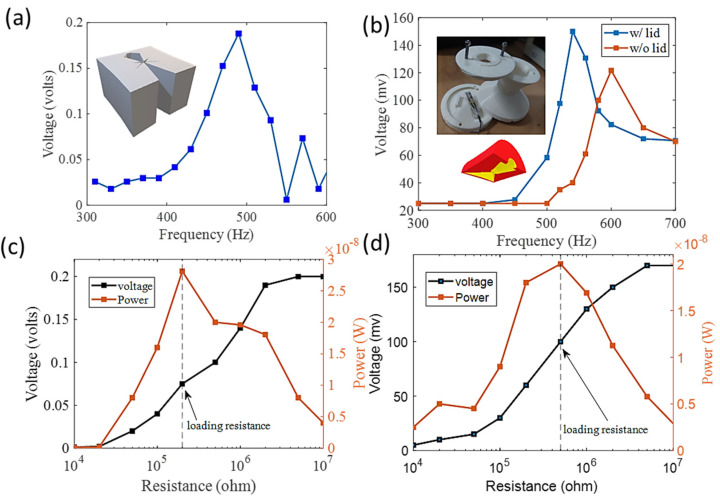
Energy harvesting results for PVDF cantilevers. (**a**,**c**) Voltage and power vs. resistance for 2.5D cavity. (**b**,**d**) Voltage and power comparison for 3D cavity (with and without lid).

As shown in Figure 6a,c, the 2.5D cavity generated a maximum open-circuit voltage of 0.2 V and a peak output power of 2.8 nW at a matching load resistance of 200 kΩ. The revolved 3D cavity was tested with and without a covering lid (Figure 6b,d). The 3D cavity with the lid generated a voltage of 0.15 V, whereas the open configuration generated 0.12 V, with output power remaining near 2 nW. The covering lid shifted the resonance to a lower frequency, close to 560 Hz, whereas removing the lid shifted the resonance upward and reduced the output voltage. These results confirm that the PVDF cantilever can convert the cavity-amplified acoustic field into electrical output, although the generated power remains in the nanowatt range.

#### 3.5.2. PZT Disc and Stack: High-Output Energy Harvesting

**Figure 7:**
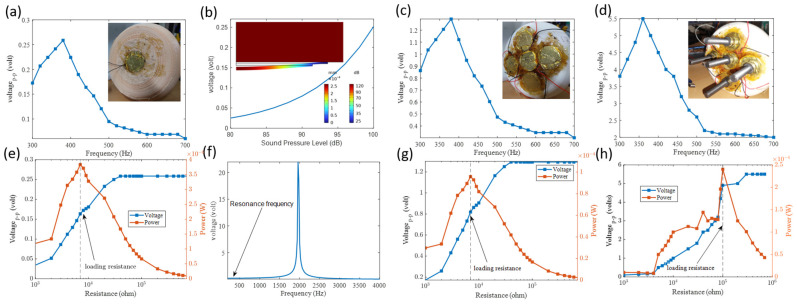
Performance of PZT-based harvesting. (**a**,**e**) Voltage and power for single PZT disc. (**b**,**f**) Simulation of PZT disc response and eigenfrequency analysis. (**c**,**g**) Results for 5-layer PZT stack. (**d**,**h**) Maximum performance achieved using 5-layer stack with 18 g tip mass (0.25 mW peak power).

To overcome the limited output power of the PVDF cantilever, the harvesting element was replaced with a lead zirconate titanate (PZT) disc with a diameter of 27 mm, which was sealed at the bottom of the cavity, as shown in Figure S3. Although the manufacturer-specified resonance frequency of the PZT disc is approximately 2000 Hz (Figure 7f), it was effectively coupled to the cavity resonance near ≤500 Hz through acoustic pressure amplification.

When integrated into the 3D cavity, a single PZT disc generated an output voltage of 0.25 V_pp_ and an output power of 3.8 μW (Figure 7a,e). The power was calculated from the voltage across the optimized load resistance using $P=V^{2}/R_{L}$. The measured output showed good agreement with the multiphysics simulation (Figure 7b,f).

A stack of five PZT discs further increased the output voltage to 1.3 V_pp_ and the output power to 90 μW (Figure 7c,g). To further enhance the electromechanical response, an 18 g tip mass was attached to the five-PZT stack to tune its mechanical resonance. This mass-loaded configuration achieved a substantial performance improvement, generating a maximum output voltage of 5.5 V_pp_ and a peak output power of 0.25 mW under 100 dB SPL (Figure 7d,h). The mass-loaded five-PZT stack exhibited its maximum electrical output at a lower frequency than the bare cavity resonance because the added mass shifted the mechanical resonance of the PZT assembly toward approximately 360 Hz.

A performance comparison between this work and previously reported acoustic energy harvesters is summarized in Table 3. The voltage response of the five-PZT cavity as a function of distance and sound pressure level is provided in Figure S4. Compared to the planar Helmholtz framework by Yuan et al. [44], which has a volume of $200\;{\mathrm{cm}}^{3}$ and generates 27.2 μW, our device delivers nearly 10 times the power in half the physical space. Similarly, compared to the massive sonic crystal framework by Yang et al. [11], which requires a volume of over $3027\;{\mathrm{cm}}^{3}$ to hit 429 μW, our device achieves a comparable power order of magnitude while being 28 times smaller in volume.

Based on ideal linear scaling, a 10×10 array of the optimized unit cells is projected to deliver approximately 25 mW. The demonstrated acoustic energy harvester offers a complementary route for localized power generation in indoor, underground, and high-noise environments where conventional solar and wind energy harvesting is limited.

### 3.6. Device Reliability and Ennvironmental Resislence

The resonance frequency of any acoustic cavity (such as Helmholtz-type resonator) is directly proportional to the speed of sound *c*. The speed of sound in air is strictly dependent on the absolute temperature *T* (in Kelvin), governed by the ideal gas relationship:

(8) $$c=\sqrt{\gamma RT}$$

where γ is the adiabatic index (1.4 for air) and *R* is the specific gas constant [49]. Because our scaled 3D resonator relies on a precise matching between the traffic noise frequency (≤500 Hz) and the internal cavity volume, a change in ambient temperature will shift the device’s acoustic resonance peak. During summer season, speed of sound increases, shifting the cavity resonance frequency upward, while it moves downwards during winter season [50]. For the lead zirconate titanate (PZT) stack, temperature shifts alter the dielectric permittivity ($\varepsilon^{T}$), the elastic compliance coefficients ($s^{E}$), and the piezoelectric charge constant ($d_{31}$ or $d_{33}$) [51]. PZT generally maintains highly stable performance well below its Curie temperature (which is typically >300 °C) [52]. Within ambient outdoor operating limits (e.g., −20 °C to 50 °C), the electromechanical coupling factors change by only a minor percentage. Acoustic energy harvesting induces incredibly low mechanical strain compared to traditional kinetic energy harvesters (like base-shakers or wind turbines) [53]. This pressure yields microscopic structural deflections in the micrometer or sub-micrometer range. Because the induced cyclic stress is orders of magnitude below the structural fatigue limits of both the PZT ceramics (∼50 MPa) and the brass substrate backing, the probability of mechanical fracture or depolarization over billions of operating cycles is exceptionally low. Since PLA is prone to degrade with ultraviolet radiation, the optimized heterogeneous geometry can seamlessly be printed using robust engineering polymers like Acrylonitrile Styrene Acrylate (ASA) or Polycarbonate (PC), or cast in weather-resistant resin to guarantee decades of structural integrity without altering the optimized internal cavity volume [54].

## 4. Conclusions

In this study, we developed an active-learning-guided inverse-design strategy for acoustic metamaterial resonators that simultaneously amplify low-frequency sound, suppress acoustic transmission, and enable piezoelectric energy harvesting. A GPR surrogate model was trained using FEM-generated cavity designs sampled from the radial-coordinate design space and was iteratively updated with FEM-validated candidates selected through GA optimization. This AL + GA strategy efficiently explored spatially heterogeneous cavity geometries and achieved high pressure amplification with substantially fewer FEM evaluations than conventional GA.

The optimized nine-point cavity, originally designed at 3.5 kHz, was geometrically scaled and converted into a 2.5D prototype, achieving a pressure amplification factor of approximately 20 near 490 Hz. To enable manufacturable 3D integration, a revolved 3D cavity was generated from the six-point optimized geometry. This 3D configuration experimentally achieved pressure amplification exceeding 30 near the target frequency and a transmission loss of approximately 14 dB, confirming its dual functionality for acoustic energy concentration and noise suppression.

For energy harvesting, the PVDF cantilever served as a proof-of-concept transducer, generating 0.2 V and 2.8 nW in the 2.5D cavity. The mass-loaded five-PZT stack substantially enhanced the electrical output, producing up to 5.5 V_pp_ and 0.25 mW under 100 dB SPL, corresponding to a normalized power density of approximately 0.58 μW Pa^−2^ cm^−3^. To the best of our knowledge, this represents one of the highest normalized power densities reported for acoustic energy harvesters under comparable input conditions. These results demonstrate the potential of active-learning-designed acoustic metamaterial resonators as multifunctional noise barriers for localized energy harvesting and self-powered sensing in high-noise environments. In future we will consider the following research directions:

- **Stochastic and Broadband Ambient Signals:** The current prototype was optimized and validated using deterministic harmonic frequency sweeps centered around 500 Hz. Future work will focus on optimizing the cavity geometries for broadband, stochastic low-frequency profiles using non-harmonic white noise or recorded field samples from highway traffic and subway corridors.
- **Scalable Multi-Unit Metamaterial Arrays:** While a single unit cell suppresses transmission by 14 dB, practical implementations require scaling up into macroscopic noise barrier panels. Future work will study the spatial acoustic coupling effects of arranging these optimized cells into a 10 ×10 array. Based on ideal linear scaling, such an array is projected to deliver approximately 25 mW, enough to drive low-power distributed edge sensors, wireless transceivers, and real-time noise decibel micro-controllers.
- **Advanced Material Lifespans:** To move past laboratory PLA prototyping, future investigations will test manufacturing avenues using carbon-fiber-reinforced composites or Polycarbonate via industrial stereolithography (SLA) printing to enhance weatherproofing, thermal drift resistance, and structural dampening stability.

We are optimistic that aforementioned research directions could pave way to built high performance acoustic energy harvesters.

## Figures and Tables

**Table 1:** Summary of the geometric optimization results targeting frequencies of 2.5, 3.0, 3.5, and 4.0 kHz. The table compares the structural evolution and resulting pressure amplification factors for four-point, six-point, and nine-point variable definitions. The highlighted row indicates the selected optimal design (nine-point geometry at 3.5 kHz) used for experimental verification.

	6 Points Optimization		4 Points Optimization		9 Points Optimization	
Frequency (Hz)	amplification	Structure	amplification	Structure	amplification	Structure
4000	**4.58**	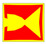	**2.03**	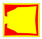	**19.41**	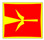
3500	6.52	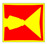	2.49	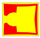	16.59	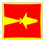
3000	8.72	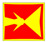	3.94	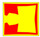	**13.94**	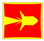
2500	**12.83**	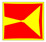	**5.39**	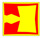		

**Table 2:** Benchmarking of conventional GA and AL + GA optimization for cavity design. The table summarizes the population size, number of generations, total FEM evaluations, and pressure amplification factors for the six-point and nine-point geometries. AL + GA achieved the same amplification as conventional GA with tenfold fewer FEM evaluations in the six-point case and achieved a higher amplification factor with fivefold fewer FEM evaluations in the nine-point case. AL assisted results are highlighted in yellow.

Points	Population	Generations	FEM Evaluations	Amplification, Scheme
6 points	20	200	4000	6.8, GA
	100	150	1500	6.8, GA
	100	4	400	6.8, AL + GA
9 points	20	150	3000	16, GA
	100	300	30,000	18, GA
	100	60	6000	20.1, AL + GA

**Table 3:** Comparison of acoustic energy harvesting performance with previously reported systems. Highlighted row shows the performance of our device.

Pressure (Pa)	Freq (Hz)	Power (μW)	Volume (cm^3^)	Power Density (μW/cm^3^)	Power Density (μW/Pa^2^cm^3^)	Reference
2	217	27.2	200	0.136	0.034	[44]
6.3	5545	429	3027.6	0.141696	0.036	[11]
2	146	2.2	1160	0.001897	0.000046	[45]
63.24	2100	49	21.2	2.311321	0.00057	[46]
2	1324	7.5	735	0.010204	0.0025	[47]
2	183	7.3	251	0.029084	0.0072	[4]
9	199	12700	840	15.11905	0.185	[48]
2	360	250	108	2.31	0.5775	This work (mass-loaded five-PZT stack)

## Data Availability

The data presented in this study are available on request from the corresponding author.

## Supplementary Materials

The following supporting information can be downloaded at: https://www.mdpi.com/article/10.3390/mi17060685/s1. Figure S1. (a) Cavity shapes possible with formulation. (b) Output prediction by machine learning model at different generations with updating. Figure S2. Simulation of 2.5D cavity design. Figure S3. Photos for the attached piezoelectric materials on the designed metamaterial cavity. (a–c) PVDF bonding with 2.5D cavity. (d–f) Shows sealing of PZT disc and weights. Figure S4. Experimental voltage measured from the five-PZT stack at different SPL values. Figure S5. Mesh convergence analysis showing the variation in the simulated pressure amplification factor with maximum element size. A maximum element size of 1 mm was selected for the final simulations because further mesh refinement produced negligible changes in the calculated response. Table S1. Piezoelectric and physical properties of PZT-5H circular plate.

## References

[1] Yin J., Wang S., Xu J., Zhao X., Chen G., Xiao X., Chen J. (2025). Leveraging giant magnetoelasticity in soft matter for acoustic energy harvesting. Matter.

[2] Münzel T., Molitor M., Kuntic M., Hahad O., Röösli M., Engelmann N., Basner M., Daiber A., Sørensen M. (2024). Transportation Noise Pollution and Cardiovascular Health. Circ. Res..

[3] Kim S., Choi J., Seung H.M., Jung I., Ryu K.H., Song H.C., Kang C.Y., Kim M. (2022). Gradient-index phononic crystal and Helmholtz resonator coupled structure for high-performance acoustic energy harvesting. Nano Energy.

[4] Yuan M., Cao Z., Luo J., Pang Z. (2018). Helix structure for low frequency acoustic energy harvesting. Rev. Sci. Instrum..

[5] Pillai M.A., Ezhilarasi D. (2016). Improved Acoustic Energy Harvester Using Tapered Neck Helmholtz Resonator and Piezoelectric Cantilever Undergoing Concurrent Bending and Twisting. Procedia Eng..

[6] Zhao L., Duan J., Liu L., Wang J., Duan Y., Vaillant-Roca L., Yang X., Tang Q. (2021). Boosting power conversion efficiency by hybrid triboelectric nanogenerator/silicon tandem solar cell toward rain energy harvesting. Nano Energy.

[7] Xu W., Guo J., Wen H., Meng X., Hong H., Yuan J., Gao J., Liu D., Ran Q., Wang Y. (2022). Laminated triboelectric acoustic energy harvester based on electrospun nanofiber towards real-time noise decibel monitoring. Nano Energy.

[8] Liu L., Yang X., Zhao L., Hong H., Cui H., Duan J., Yang Q., Tang Q. (2021). Nodding Duck Structure Multi-track Directional Freestanding Triboelectric Nanogenerator toward Low-Frequency Ocean Wave Energy Harvesting. ACS Nano.

[9] Feng Y., Zhang L., Zheng Y., Wang D., Zhou F., Liu W. (2019). Leaves based triboelectric nanogenerator (TENG) and TENG tree for wind energy harvesting. Nano Energy.

[10] Noh S., Lee H., Choi B. (2013). A study on the acoustic energy harvesting with Helmholtz resonator and piezoelectric cantilevers. Int. J. Precis. Eng. Manuf..

[11] Yang A., Li P., Wen Y., Lu C., Peng X., Zhang J., He W. (2013). Enhanced acoustic energy harvesting using coupled resonance structure of sonic crystal and helmholtz resonator. Appl. Phys. Express.

[12] Yaw Z., Zhang Y., Liu C., Chen Z., Ni Y.-Q., Lai S.-K. (2025). Reconfigurable 3D-printed 1-bit coding metasurface for simultaneous acoustic focusing and energy harvesting at low-frequency regime. Nano Energy.

[13] Deng T., Zhao L., Jin F. (2025). Trampoline metamaterial coupled with Helmholtz resonator for enhanced acoustic piezoelectric energy harvesting. Appl. Math. Model..

[14] Li D., Hu M., Wu F., Liu K., Gao M., Ju Z., Zhao J., Bao A. (2022). Design of tunable low-frequency acoustic energy harvesting barrier for subway tunnel based on an optimized Helmholtz resonator and a PZT circular plate. Energy Rep..

[15] Wang Y., Zhu X., Zhang T., Bano S., Pan H., Qi L., Zhang Z., Yuan Y. (2018). A renewable low-frequency acoustic energy harvesting noise barrier for high-speed railways using a Helmholtz resonator and a PVDF film. Appl. Energy.

[16] Zhang M., Zhong W., Zhang X. (2012). Defect-free localized modes and coupled-resonator acoustic waveguides constructed in two-dimensional phononic quasicrystals. J. Appl. Phys..

[17] Zhou S., Jia C., Shu G., Guan Z., Wu H., Li J., Ou-Yang W. (2024). Recent advances in TENGs collecting acoustic energy: From low-frequency sound to ultrasound. Nano Energy.

[18] Jin M., Liang B., Yang J., Yang J., Cheng J.-C. (2019). Ultrathin planar metasurface-based acoustic energy harvester with deep subwavelength thickness and mechanical rigidity. Sci. Rep..

[19] Cui X.B., Huang C.P., Hu J.H. (2015). Sound energy harvesting using an acoustic grating. J. Appl. Phys..

[20] Vocca H., Neri I., Travasso F., Gammaitoni L. (2012). Kinetic energy harvesting with bistable oscillators. Appl. Energy.

[21] Cao Y., Shao H., Wang H., Li X., Zhu M., Fang J., Cheng T., Lin T. (2022). A full-textile triboelectric nanogenerator with multisource energy harvesting capability. Energy Convers. Manag..

[22] Chen F., Wu Y., Ding Z., Xia X., Li S., Zheng H., Diao C., Yue G., Zi Y. (2019). A novel triboelectric nanogenerator based on electrospun polyvinylidene fluoride nanofibers for effective acoustic energy harvesting and self-powered multifunctional sensing. Nano Energy.

[23] Pundir A., Gupta A., Nag S. (2024). Multi-functional programmable active acoustic meta-device: Acoustic switch, lens, and barrier. Sci. Rep..

[24] Rani G.M., Wu C.M., Motora K.G., Umapathi R., Jose C.R.M. (2023). Acoustic-electric conversion and triboelectric properties of nature-driven CF-CNT based triboelectric nanogenerator for mechanical and sound energy harvesting. Nano Energy.

[25] Song C., Ma X., Zhao J., Zhang J., Yang F., Pan Y., Zhang X. (2022). Broadband Sound Absorption and Energy Harvesting by a Graded Array of Helmholtz Resonators. IEEE Trans. Dielectr. Electr. Insul..

[26] Iftikhar A., Hassan A., Anjum M.U., Malik S., Ali T. (2019). Ambient Acoustic Energy Harvesting using Two Connected Resonators with Piezoelement for Wireless Distributed Sensor Network. Acoust. Phys..

[27] Lee H.Y., Choi B. (2013). A multilayer PVDF composite cantilever in the Helmholtz resonator for energy harvesting from sound pressure. Smart Mater. Struct..

[28] Kumar A., Sharma A., Kumar R., Vaish R. (2018). Finite Element Study on Acoustic Energy Harvesting Using Lead-Free Piezoelectric Ceramics. J. Electron. Mater..

[29] Ma K., Tan T., Yan Z., Liu F., Liao W.-H., Zhang W. (2021). Metamaterial and Helmholtz coupled resonator for high-density acoustic energy harvesting. Nano Energy.

[30] Zhu G., Zhou Y., Si Z., Cheng Y., Wu F., Wang H., Pan Y., Xie J., Li C., Chen A. (2023). A multi-hole resonator enhanced acoustic energy harvester for ultra-high electrical output and machine-learning-assisted intelligent voice sensing. Nano Energy.

[31] Zou X., Pan J., Sun Z., Wang B., Jin Z., Xu G., Yan F. (2021). Machine learning analysis and prediction models of alkaline anion exchange membranes for fuel cells. Energy Environ. Sci..

[32] Donda K., Brahmkhatri P., Zhu Y., Dey B., Slesarenko V. (2025). Machine learning for inverse design of acoustic and elastic metamaterials. Curr. Opin. Solid State Mater. Sci..

[33] Huang J., Chen J., Mai H., Wan H., Chen R., He T. (2025). Performance prediction and inverse design of cylindrical plate-type acoustic metamaterials based on deep learning. Appl. Acoust..

[34] Dong F., Qi X., Zhao X., Li Y., Li B. (2025). Physics-guided machine learning for identifying fatigue damage in composite laminates based on acoustic emission. Compos. Struct..

[35] Shah K.A., Halim Z., Anwar S., Hsu C.-C., Rida I. (2026). Multi-sensor data fusion for smart healthcare: Optimizing specialty-based classification of imbalanced EMRs. Inf. Fusion.

[36] Lee S., Choi W., Park J.W., Kim D.S., Nahm S., Jeon W., Gu G.X., Kim M., Ryu S. (2022). Machine learning-enabled development of high performance gradient-index phononic crystals for energy focusing and harvesting. Nano Energy.

[37] Pennec Y., Jin Y., Djafari-Rouhani B. (2019). Phononic and photonic crystals for sensing applications. Adv. Appl. Mech..

[38] Qian X., Yang R. (2021). Machine learning for predicting thermal transport properties of solids. Mater. Sci. Eng. R Rep..

[39] Jin Y., He L., Wen Z., Mortazavi B., Guo H., Torrent D., Djafari-Rouhani B., Rabczuk T., Zhuang X., Li Y. (2022). Intelligent on-demand design of phononic metamaterials. Nanophotonics.

[40] Nanthakumar S.S., Zhuang X., Park H.S., Nguyen C., Chen Y., Rabczuk T. (2019). Inverse design of quantum spin hall-based phononic topological insulators. J. Mech. Phys. Solids.

[41] Khatib O., Ren S., Malof J., Padilla W.J. (2021). Deep learning the electromagnetic properties of metamaterials—A comprehensive review. Adv. Funct. Mater..

[42] Miao X.-B., Dong H.W., Wang Y.-S. (2023). Deep learning of dispersion engineering in two-dimensional phononic crystals. Eng. Optim..

[43] Quinteros L., Meruane V., Flores E.I.S. (2026). Band gap scalability in optimised phononic crystals. Wave Motion.

[44] Yuan M., Cao Z., Luo J., Pang Z. (2018). Low frequency acoustic energy harvester based on a planar Helmholtz resonator. AIP Adv..

[45] Li B., Laviage A.J., You J.H., Kim Y.-J. (2013). Harvesting low-frequency acoustic energy using quarter-wavelength straight-tube acoustic resonator. Appl. Acoust..

[46] Khan F.U., Izhar I. (2016). Hybrid acoustic energy harvesting using combined electromagnetic and piezoelectric conversion. Rev. Sci. Instrum..

[47] Peng X., Wen Y., Li P., Yang A., Bai X. (2013). A wideband acoustic energy harvester using a three degree-of-freedom architecture. Appl. Phys. Lett..

[48] Li B., You J.H., Kim Y.-J. (2013). Low frequency acoustic energy harvesting using PZT piezoelectric plates in a straight tube resonator. Smart Mater. Struct..

[49] Kinsler L.E., Frey A.R., Coppens A.B., Sanders J.V. (2000). Fundamentals of Acoustics.

[50] Cox T., d’Antonio P. (2016). Acoustic Absorbers and Diffusers: Theory, Design and Application.

[51] Gautschi G. (2002). Piezoelectric sensors. Piezoelectric Sensorics: Force Strain Pressure Acceleration and Acoustic Emission Sensors Materials and Amplifiers.

[52] Chen Z., Hao Y., Huang J., Zhou Z., Li Y., Liang R. (2023). Poling above the Curie temperature driven large enhancement in piezoelectric performance of Mn doped PZT-based piezoceramics. Nano Energy.

[53] Erturk A., Inman D.J. (2011). Piezoelectric Energy Harvesting.

[54] Kutz M. (2011). Applied Plastics Engineering Handbook: Processing and Materials.

