# Supplementary material for: Active-Learning-Guided Acoustic Metamaterial Resonators for Low-Frequency Noise Suppression and Piezoelectric Energy Harvesting
Source: Micromachines (Basel). 2026 May 31;17(6):685. doi: 10.3390/mi17060685 (PMC13302984; doi:10.3390/mi17060685)
Supplement: Supplementary file 1 [file micromachines-17-00685-s001.zip › micromachines-4314817-supplementary.pdf]

[Supplementary Material]

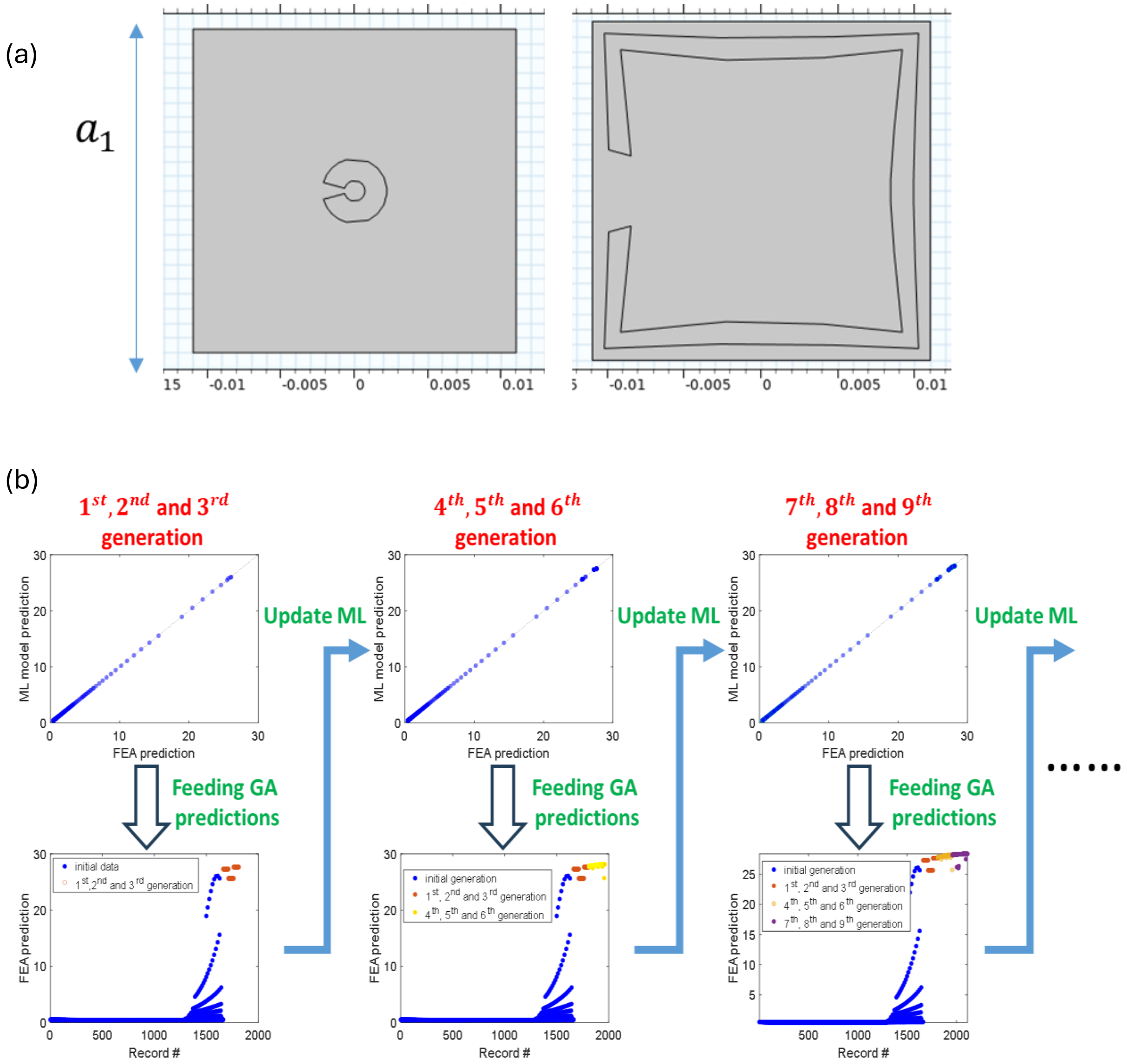


**Figure S1.(a)** Cavity shapes possible with formulation.(b)Output prediction by machine learnin g model at different generations with updating.

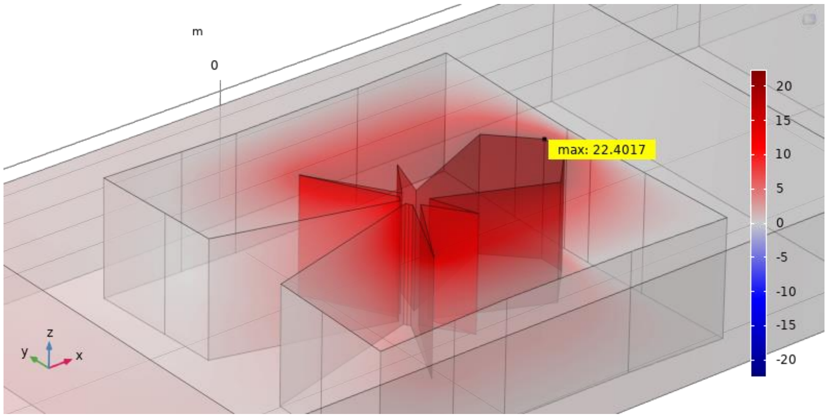


**Figure S2.** Simulation of 2.5D cavity design

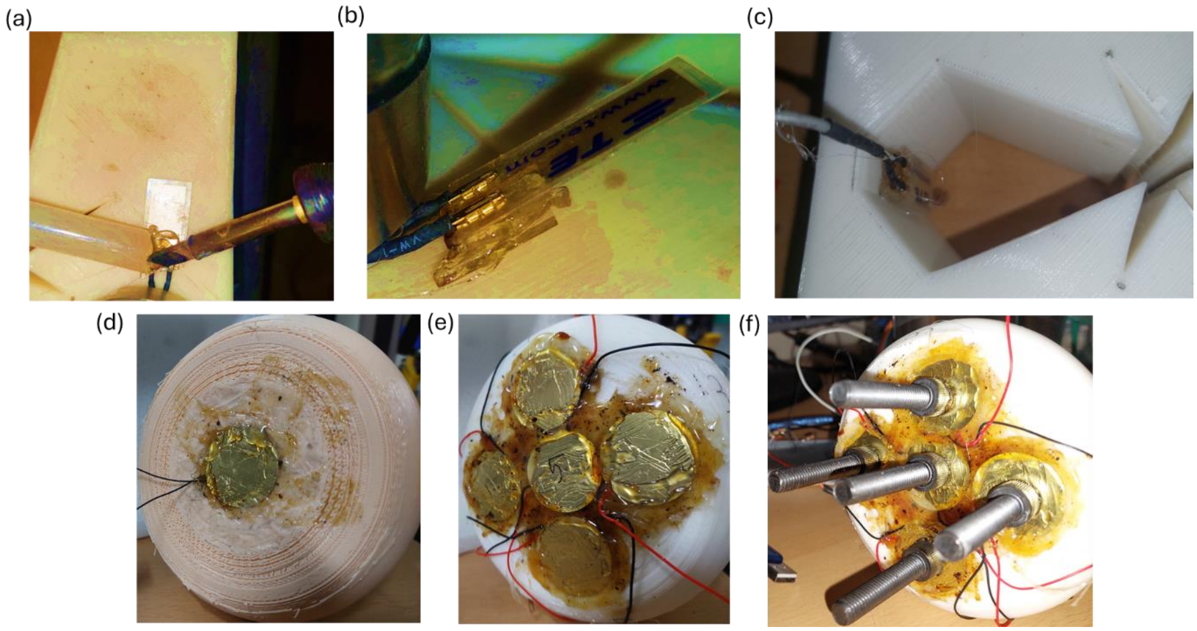


**Figure S3.** Photos for the attached piezoelectric materials on the designed metamaterial cavity. (a-c) PVDF bonding with 2.5D cavity. (d-f) Shows sealing of PZT disc and weights.

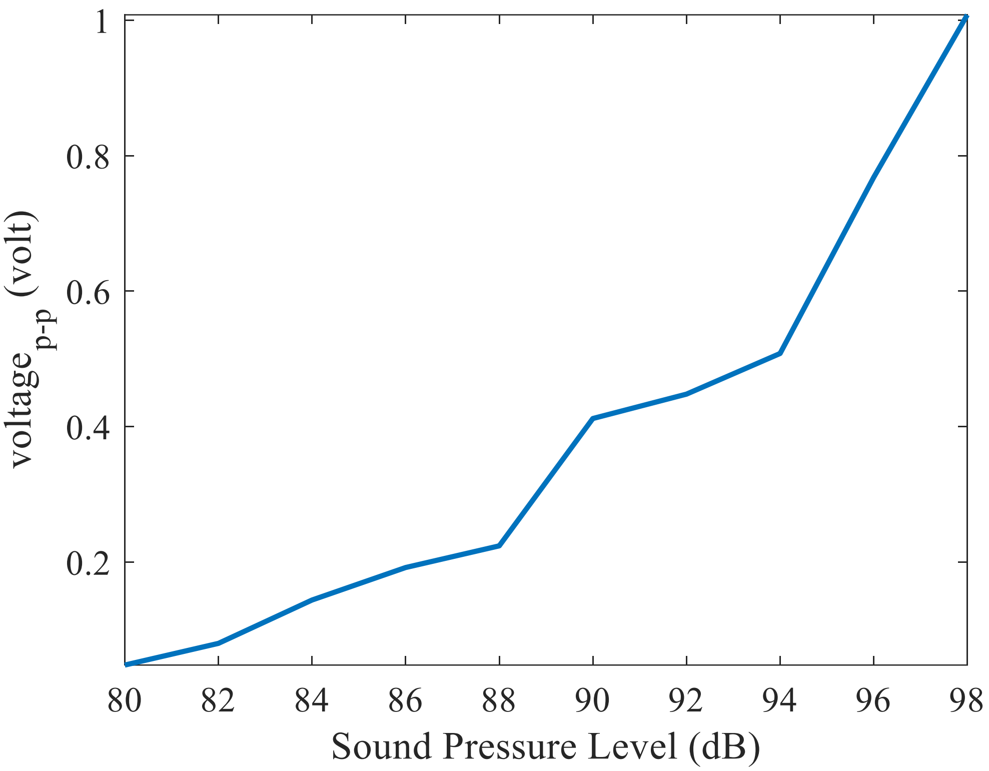


**Figure S4.** Experimental voltage measured from the five-PZT stack at different SPL values.

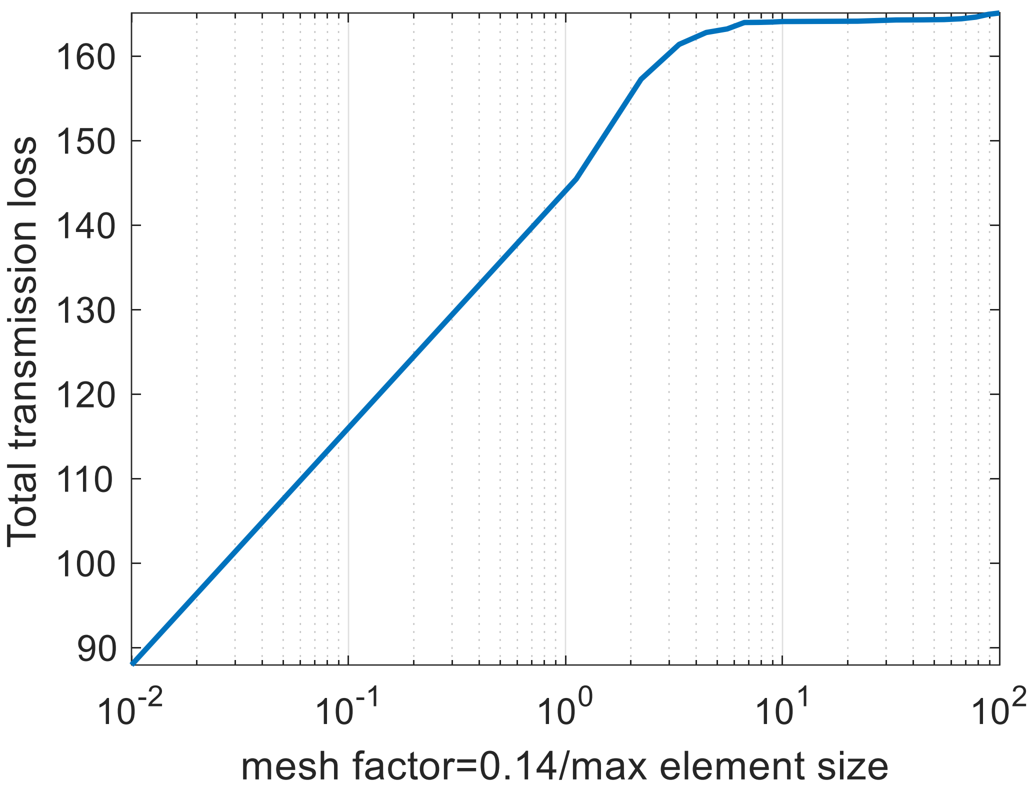


**Figure S5.** Mesh convergence analysis showing the variation in the simulated pressure amplification factor with maximum element size. A maximum element size of 1 mm was selected for the final simulations because further mesh refinement produced negligible changes in the calculated response.

**Table S1.** Piezoelectric and physical properties of PZT-5H circular plate

| Parameter | Value |
|---|---|
| Brass Radius | 13mm |
| PZT radius | 10mm |
| Poission ratio | 0.23 |
| Thickness | 0.2mm |
| Density | $7500 kg/m^3$ |
